# Tris (2,3-Dibromopropyl) Isocyanurate (TDBP-TAZTO or TBC) Shows Different Toxicity Depending on the Degree of Differentiation of the Human Neuroblastoma (SH-SY5Y) Cell Line

**DOI:** 10.1007/s12640-021-00399-x

**Published:** 2021-08-03

**Authors:** Konrad A. Szychowski, Bartosz Skóra, Marzena Mańdziuk

**Affiliations:** 1 Department of Biotechnology and Cell Biology, Medical College, University of Information Technology and Management in Rzeszow, Sucharskiego 2, 35-225 Rzeszow, Poland; 2 Department of Physiotherapy, Medical College, University of Information Technology and Management in Rzeszow, Sucharskiego 2, 35-225 Rzeszow, Poland

**Keywords:** TDBP-TAZTO, TBC, Flame retardant, Neuron, Differentiation, Retinoic acid

## Abstract

Tris (2,3-dibromopropyl) isocyanurate (TDBP-TAZTO or TBC) is a heterocyclic hexabromated flame retardant. It is widely used during the production of many synthetic compounds. High concentrations of TDBP-TAZTO were found in river water, surface sediments, soil, earthworms, and carp tissues. Moreover, it has been shown that this compound can cross the blood–brain barrier and accumulate in the gut and brain of carp. The aryl hydrocarbon receptor (AhR) has been characterized as a multifunctional intracellular sensor and receptor. AhR is an activator of cytochrome P450 1A1 and 1A2, which metabolize various toxic compounds. The aim of the study was to explain how/whether TDBP-TAZTO increases the expression and/or activity of the CYP1A1 enzyme and the *AhR* and *TUBB3* expression during SH-SY5Y cell differentiation. SH-SY5Y cells were differentiated for 7 and 14 days using retinoic acid. Cell viability, ethoxyresorufin-O-deethylase (EROD) activity, and mRNA expression of *CYP1A1*, *AhR*, and *TUBB3* were assessed. Our experiment showed that, during the differentiation process, the ability of TDBP-TAZTO to induce EROD activity in SH-SY5Y cells subsequently decreased, which may have been an effect of cell differentiation into neurons. Moreover, the results suggest that TDBP-TAZTO can affect the differentiation process. Since no *CYP2B6* mRNA expression was detected, the CAR receptor may not be involved in the TDBP-TAZTO mechanism of action. However, more research is needed in this field to elucidate this mechanism precisely.

## Introduction

Tris (2,3-dibromopropyl) isocyanurate, known by its abbreviations TBC or TDBP-TAZTO, is a heterocyclic hexabromated flame retardant. It was developed as a substitute for tetrabromobisphenol A (TBBPA) and other brominated flame retardants (BFRs), which exhibit significant endocrine-disrupting properties, hepatoxicity, immunotoxicity, and neurotoxicity (Alzualde et al. 2018; Honkisz and Wójtowicz 2015; Tanaka et al. 2018; Wojtowicz et al. 2014). Due to its stability, TDBP-TAZTO is widely used in polyolefins, polyphenylalkenes, unsaturated polyester, synthetic rubber, and synthetic fibers (Zhu et al. 2012). The first described the use of TDBP-TAZTO dates back to 1959 when it was used in styrene as a flame retardant (Dong et al. 2015). The annual TDBP-TAZTO production in China in the 1990s was estimated at over 500 metric tons (Li et al. 2011). Following the cessation of the commercial production of some BFRs and the toxicity of some of these compounds, an increase in TDBP-TAZTO production and use can be expected worldwide, including Europe, the USA, and Japan (Zhu et al. 2012).

TDBP-TAZTO was first detected in 2009 in environmental samples from Hunan Province (China) (Ruan et al. 2009). High concentrations of TDBP-TAZTO were found in river water (2.33–163 ng/L), surface sediments (85.0–6029 ng/g), soil (19.6–672 ng/g), earthworms (9.75–78.8 ng/g), and carp tissues (12.0–646 ng/g) (Ruan et al. 2009). Moreover, TDBP-TAZTO has been shown to be able to cross the blood–brain barrier (BBB) and accumulate in the gut and brain of carp (Ruan et al. 2009). Therefore, TDBP-TAZTO is now considered to be a persistent compound with strong potential for accumulation in the organisms (Ruan et al. 2009; Zhu et al. 2012). Studies carried out on zebrafish embryos (*Danio rerio*) have shown that TDBP-TAZTO can cause potential toxic effects by disrupting the development and function of the endocrine system, damaging cell mitochondria, and influencing the development and function of the swim bladder (Zhang et al. 2011; Li et al. 2011). Other research groups have shown that TDBP-TAZTO can induce significant toxicity in murine organs, especially the liver and lungs (Li et al. 2015). Currently, the toxicity of TDBP-TAZTOs has been well characterized in relation to various organisms in vivo. Unfortunately, the molecular mechanism of the TDBP-TAZTO action has not been elucidated. Some research groups see a key role of oxidative stress in the TDBP-TAZTO mechanism of action (Dong et al. 2015). However, further research is needed to understand this mechanism.

The only available studies showed that TDBP-TAZTO administered daily for 6 months at doses of 5 or 50 mg/kg caused cognitive impairment and depressive-like behavior in adult male rats. Furthermore, TDBP-TAZTO induced significant neurotoxicity in hippocampal cells, characterized by an increase in markers of inflammation and oxidative stress and an increase in cellular apoptosis in the hippocampus (Ye et al. 2015). In vitro studies demonstrated that TDBP-TAZTO was toxic at concentrations of 5–10 µM in primary cultures of the neurons of developing cerebellar granule cells but not in mature cerebellar granule neurons in rats (Qu et al. 2011). Moreover, TDBP-TAZTO at concentrations of 12.5, 25, and 50 µM damaged human neuroblastoma cells SH-SY5Y cells and led to apoptosis and severe oxidative stress (Dong et al. 2015).

The aryl hydrocarbon receptor (AhR) has been characterized as a multifunctional intracellular sensor and receptor (Bock 2020). AhR is an activator of cytochrome P450 1A1 (CYP1A1) and CYP1A2 (Beedanagari et al. 2009). CYP1A1 metabolizes various toxic and environmental carcinogens and then converts procarcinogenic compounds into carcinogenic metabolites (Androutsopoulos et al. 2009; Ye et al. 2019). β‐Naphthoflavone (βNF) and 2,3,7,8‐tetrachlorodibenzo‐p‐dioxin (TCDD) activate AhR and initiate its binding to the xenobiotic responsive element (XRE) and, consequently, upregulate the expression of CYP1A1 (Kobayashi et al. 1996; Ye et al. 2019). Many xenobiotics similar to AhR activate the constitutive androstane receptor (CAR), which in response upregulates the *CYP2B6* mRNA expression (Rhodes et al. 2011). Therefore, we decided to measure the mRNA expression of *CYP2B6* in our study to find out whether TDBP-TAZTO is a potential CAR agonist as well*.*

To date, the knowledge of the impact of TDBP-TAZTO on the induction of the neuron differentiation process is still insufficient. Moreover, the correlation between TDBP-TAZTO and the time of treatment during the neuron differentiation process has never been shown. According to literature, tubulin beta 3 class III (*TUBB3*) genes significantly increase in differentiated SH-SY5Y cells and are also present in mature neurons (Latremoliere et al. 2018). For this reason, the *TUBB3* gene has been chosen as a marker of differentiation to mature neuronal cells (Constantinescu et al. 2007; Kovalevich and Langford 2013). These aspects are important due to the potential neuromodulatory effect of TDBP-TAZTO in in vivo research, its engagement in the neurosecretory functions of animal brains, and its increasing accumulation in the environment.

Therefore, the aim of the study was to determine whether TDBP-TAZTO was able to affect the neuron differentiation process on the 7th and 14th day after application and whether it influenced the expression and/or activity of the CYP1A1, CYP1A2, and CYP2B6 enzymes and the *AhR* and *TUBB3* expression during SH-SY5Y cell differentiation.

## Material and Methods

### Reagents

Dulbecco’s phosphate-buffered saline without calcium and magnesium (DPBS) and DMEM/F12 without phenol red (16–405-CV) were purchased from Corning (Manassas, VA, USA). CAY10464 (1,3-dichloro-5-[(1E)-2-(4-methoxyphenyl)ethenyl]-benzene) and ethoxyresorufin-O-deethylase (EROD) substrate were purchased from Cayman Chemical (Michigan, USA). β-Naphthoflavone (βNF), 2′,7′-dichlorofluorescin diacetate (H_2_DCFDA), trypsin, penicillin, streptomycin, glycerol, 4-(2-hydroxyethyl)-1-piperazineethanesulfonic acid (HEPES), 3-[(3-cholamidopropyl)dimethylammonio]-1-propanesulfonate hydrate (CHAPS), dithiothreitol (DTT), NaCl, ethylenediaminetetraacetic acid (EDTA), tris (2, 3-dibromopropyl) isocyanurate (TDBP-TAZTO or TBC) (269,999), all-trans retinoid acids (R2500), and dimethyl sulfoxide (DMSO) were purchased from Sigma-Aldrich (St. Louis, MO, USA). The substrate for caspase-3 (235,400) was purchased from Merck (Darmstadt, Germany). Fast Probe qPCR Master Mix and heat-inactivated fetal bovine serum (FBS) were purchased from EURx (Gdańsk, Poland). The High Capacity cDNA Reverse Transcription Kit and TaqMan® probes corresponding to specific genes encoding *ACTB* (Hs01060665_g1), *AhR* (Hs00169233_m1), *CYP1A1* (Hs01054796_g1), *CYP1A2* (Hs00167927_m1), *CYP2B6* (Hs04183483_g1), and *TUBB3* (Hs00801390_s1) were obtained from Life Technologies Applied Biosystems (Foster City, CA, USA). Stock solutions of the TDBP-TAZTO were prepared in DMSO and were added to the DMEM/F12 medium. The final concentration of DMSO in the culture medium was always 0.1%.

### SH-SY5Y Cell Culture, Differentiation, and Treatment

Cells from the human neuroblastoma (SH-SY5Y) cell line were obtained from the American Type Culture Collection (ATCC distributor: LGC Standards, Łomianki, Poland). The SH-SY5Y cells were maintained in DMEM/F12 medium supplemented with 10% heat-inactivated FBS at 37 °C in a humidified atmosphere with 5% CO_2_. The cells were seeded in 96-well culture plates at a density of 6 × 10^3^ cells per well for 24-h treatment and initially cultured before the experiment for 24 h. Subsequently, the medium was replaced with a fresh one by increasing the concentrations (1, 10, 50, and 100 nM; and 1, 10, 50, and 100 µM) of TDBP-TAZTO for 24 h. The process of SH-SY5Y differentiation was induced by maintaining the cells in DMEM/F12 without phenol red supplemented with 1% FBS and 10 µM all-trans retinoic acid (RA). According to the literature, the minimal time of differentiation of SH-SY5Y is 7 days (Zhang et al. 2021). However, the overwhelming majority of papers report that SH-SY5Y cells are not fully differentiated until day 14 (Schneider et al. 2011; Shipley et al. 2016). Therefore, we chose both time frame intervals in our study to obtain information on whether there is a difference between them. The medium was replaced with a new one (DMEM/F12, 1% FBS, and 10 µM RA) after every 2 days up to the 7th or 14th day of differentiation. Subsequently, on the 7th or 14th day of differentiation, the medium was replaced with a fresh one, containing increasing concentrations (1, 10, 50, and 100 nM; and 1, 10, 50, and 100 µM) of TDBP-TAZTO for 24 h. Based on the obtained data, the concentration of 1 µM TDBP-TAZTO was selected for further analyses. Moreover, 1 µM beta-naphthoflavone (βNF), i.e., an agonist of AhR, and the 1 µM CAY10464 selective antagonist of AhR were used (tool compounds).

### Lactate Dehydrogenase (LDH) Cytotoxicity Assay

The cytotoxicity detection kit is a colorimetric assay for quantification of cell death and cell lysis based on the release of LDH from the cytosol of damaged cells into the surrounding medium (Koh and Choi 1987). An increase in the amount of dead or plasma membrane-damaged cells results in an increase in LDH activity in the culture medium. After treating the cells with TDBP-TAZTO or TDBP-TAZTO with the tool compound, the culture supernatants were collected and incubated in a reaction mixture from the kit. After 30 min, the reaction was stopped by adding 1 N HCl, and absorbance at a wavelength of 490 nm was measured using the FilterMax F5 Multi-Mode microplate reader (Molecular Devices, Corp., Sunnyvale, CA, USA).

### Resazurin Reduction Assay

Resazurin is a water-soluble dye that can be applied in various in vitro cell studies, and the resazurin reduction cell viability assay is considered as an alternative to the MTT assay. The method is based on the detection of metabolic activity. Moreover, the dye is not toxic to cells and facilitates continuous monitoring of the cell culture. The assay was performed with a method described previously (Szychowski et al. 2017a). On the day of analysis, a working solution of 60 µM resazurin was prepared in medium with 1% FBS. After 24 h of treatment of the cells with studied compounds, the medium in the wells was replaced with the working solution of resazurin (100 µL), and the cells were incubated at 37 °C. Fluorescence was measured at 530 nm excitation and 590 nm emission wavelengths using a FilterMax F5 Multi-Mode microplate reader (Molecular Devices, Corp., Sunnyvale, CA, USA) for 30 min and 1 and 2 h after the addition of the dye.

### Reactive Oxygen Production (ROS)

The 5 μM H_2_DCFDA was applied to determine the ability of the tested compounds to induce ROS production in the cells. In accordance with a previously described method, the cells were incubated with H_2_DCFDA in serum-free and phenol red-free medium for 45 min before the treatment with the studied compounds (Szychowski et al. 2016). After incubating the cells for 24 h with TDBP-TAZTO or TDBP-TAZTO with the tool compounds (5% CO_2_ at 37 °C), the culture medium was replaced with a fresh one to remove extracellular residual dichlorodihydrofluorescein (DCF). DCF fluorescence was measured using a microplate reader (FilterMax F5) at the maximum excitation and emission spectra of 485 and 535 nm, respectively.

### Caspase-3 Activity

Caspase-3 activity was used as a marker of cell apoptosis and was assessed as in Nicholson et al. (1995). Cells cultured with the increasing concentrations of the tested compounds or tool compounds were lysed using lysis buffer (50 mM HEPES, pH 7.4, 100 mM NaCl, 0.1% CHAPS, 1 mM EDTA, 10% glycerol, and 10 mM DTT) at 10 °C for 10 min. The lysates were incubated in caspase-3 substrate Ac-DEVD-pNA at 37 °C. Cells treated with 1 μM staurosporine were used as a positive control (results not shown). After 30 min, the absorbance of the lysates was measured at 405 nm using a FilterMax F5 Multi-Mode microplate reader. The amount of the colorimetric product was monitored continuously for 120 min. The data were analyzed using Multi-Mode Analysis software (Molecular Devices, Corp., Sunnyvale, CA, USA) and normalized to the absorbance in the vehicle-treated cells (control).

### CYP450 Activities: EROD Assay

We estimated the activity of the CYP1A1/CYP1B1 enzymes using the fluorometric ethoxyresorufin-O-deethylase (EROD) substrate. The fluorescence EROD assay was performed according to the method proposed by Kennedy et al. (1993). Briefly, the cells were seeded on 12-well plates and initially cultured for 24 h. The EROD activity was measured after the 24-h exposure to 1 µM βNF, 1 µM CAY10464, and 1 µM TDBP-TAZTO or in co-treatment with TDBP-TAZTO and the AhR agonist and antagonist. To perform the EROD assay, lysed cells were transferred into multiwell plates, and the fluorescent product resorufin was quantified within the wells with a fluorescence plate reader (FilterMax F5) at an excitation wavelength of 530 nm and an emission wavelength of 590 nm. The protein concentration was determined spectrophotometrically in triplicate for each sample at 280 nm using the ND/1000 UV/Vis Thermo Fisher NanoDrop device.

### Real-Time PCR Analysis of mRNAs Specific to Genes Encoding AhR, CYP1A1, CYP1A2, CYP2B6, and TUBB3

The experiment was conducted with a procedure described previously (Szychowski et al. 2017b). For the real-time PCR assay, SH-SY5Y cells were seeded onto 12-well plates and initially cultured for 24 h in groups of undifferentiated cells or differentiated for 7 or 14 days according to the previously described protocol. After the 24-h exposure to 1 µM TDBP-TAZTO, the samples were collected, and total RNA was extracted from the SH-SY5Y cells using an RNA isolation kit (EURx, Gdańsk, Polska) according to the manufacturer’s instructions. Moreover, cell differentiation progress was evaluated by comparison of tubulin (*TUBB3*) gene expression between control cells at time 0 and after differentiation for 7 and 14 days. Both the quality and quantity of the RNA were determined spectrophotometrically at 260 and 280 nm, respectively (ND/1000 UV/Vis; Thermo Fisher NanoDrop, USA). Two-step real-time reverse transcription (RT)-PCR was conducted with both the RT reaction and the quantitative PCR (qPCR) run using the CFX Real Time System (BioRad, USA). The RT reaction was carried out at a final volume of 20 μL with 800 ng RNA (as a cDNA template) using the cDNA reverse transcription kit in accordance with the manufacturer’s instructions. Products of the RT reaction were amplified using the fast probe qPCR master mix (EURx) with TaqMan probes as primers for specific genes encoding *ACTB*, *AhR*, *CYP1A1*, *CYP1A2*, *CYP2B6*, and *TUBB3*. The amplification was carried out in a total volume of 20 μL containing 1 × fast probe qPCR master mix (EURs) and 1.0 μL of the RT product, which was used as the PCR template. The standard qPCR procedures were carried out as follows: 2 min at 50 °C and 10 min at 95 °C, followed by 45 cycles of 15 s at 95 °C and 1 min at 60 °C. The threshold value (Ct) for each sample was set during the exponential phase, and the ΔΔCt method was used for data analysis. *ACTB* was used as the reference gene.

### Statistical Analysis

The data are presented as means ± SD. Each experiment was repeated three times independently and measured in 6 replicates (total number of replicates *n* = 18). The data were analyzed with a one-way analysis of variance (ANOVA) followed by post hoc Tukey’s test using GraphPad Prism 8.0 Statistical Analysis Panel. Significant differences were marked as follows: **p < 0.001, **p < 0.01, and *p < 0.05 vs. the control group; ##*p* < 0.01 and ###*p* < 0.001 vs. the TCS-exposed group.

## Results

### Dose–Response Analysis

#### LDH Cell Viability Assay

**Fig. 1:**
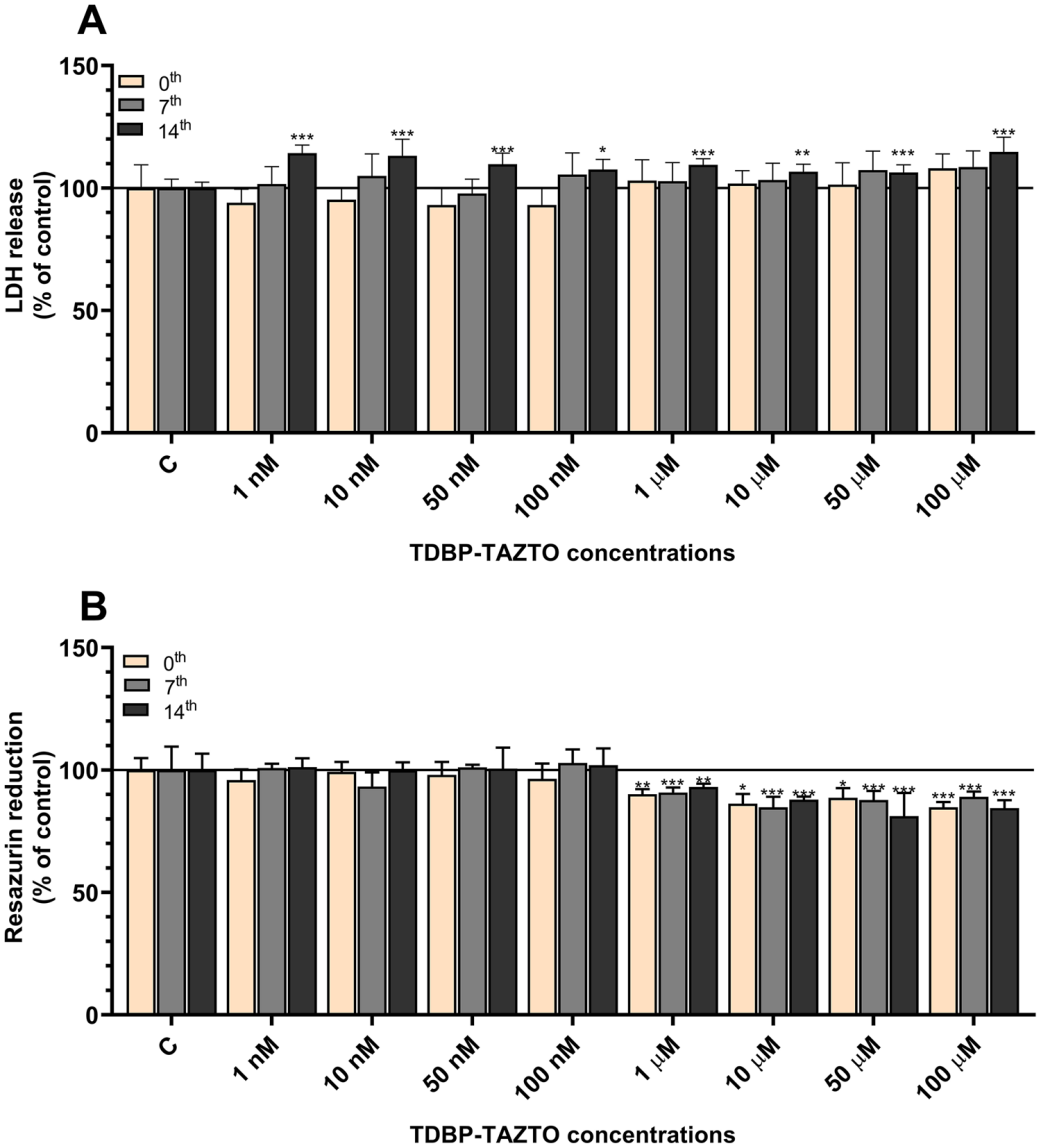
Effect of the increasing concentrations (1 nM–100 μM) of TDBP-TAZTO on LDH release **A** and resazurin reduction activity **B** after 24-h exposure of undifferentiated (time 0) human neuroblastoma SH-SY5Y cells and those differentiated for 7 or 14 days. Data are expressed as mean ± SD of three independent experiments, each of which comprised six replicates per treatment group. *p < 0.05, **p < 0.01, ***p < 0.001 vs. control cells

Our results showed that TDBP-TAZTO at all studied concentrations (1–100 nM and 1–100 µM) after the 24-h exposure of the SH-SY5Y cells (undifferentiated and differentiated for 7 days) did not increase the LDH release (Fig. 1A). In the SH-SY5Y cells differentiated for 14 days, all the concentrations of TDBP-TAZTO (1–100 nM and 1–100 µM) were found to increase the LDH release after the 24-h exposure in the range from 9.66 to 14.69%, compared to the control (Fig. 1A).

#### Resazurin Reduction Cell Viability Assay

Our results showed that TDBP-TAZTO at the concentrations of 1, 10, 50, and 100 µM decreased resazurin reduction in the undifferentiated cells and those differentiated for 7 and 14 days. In the undifferentiated cells exposed to TDBP-TAZTO for 24 h, the SH-SY5Y cell metabolism decreased by 9.86, 13.78, 11.35, and 15.29%, compared to the control (Fig. 1B). Decreased SH-SY5Y cell metabolism (by 9.28, 15.22, 12.28, and 10.93%) was detected in cells differentiated for 7 days after the 24-h exposure to TDBP-TAZTO, compared to the control (Fig. 1B). A 6.93, 12.10, 18.85, and 15.61% decrease in SH-SY5Y cell metabolism was recorded in cells differentiated for 14 days after the 24-h exposure to TDBP-TAZTO, compared to the control (Fig. 1B).

#### Reactive Oxygen Production (ROS)

**Fig. 2:**
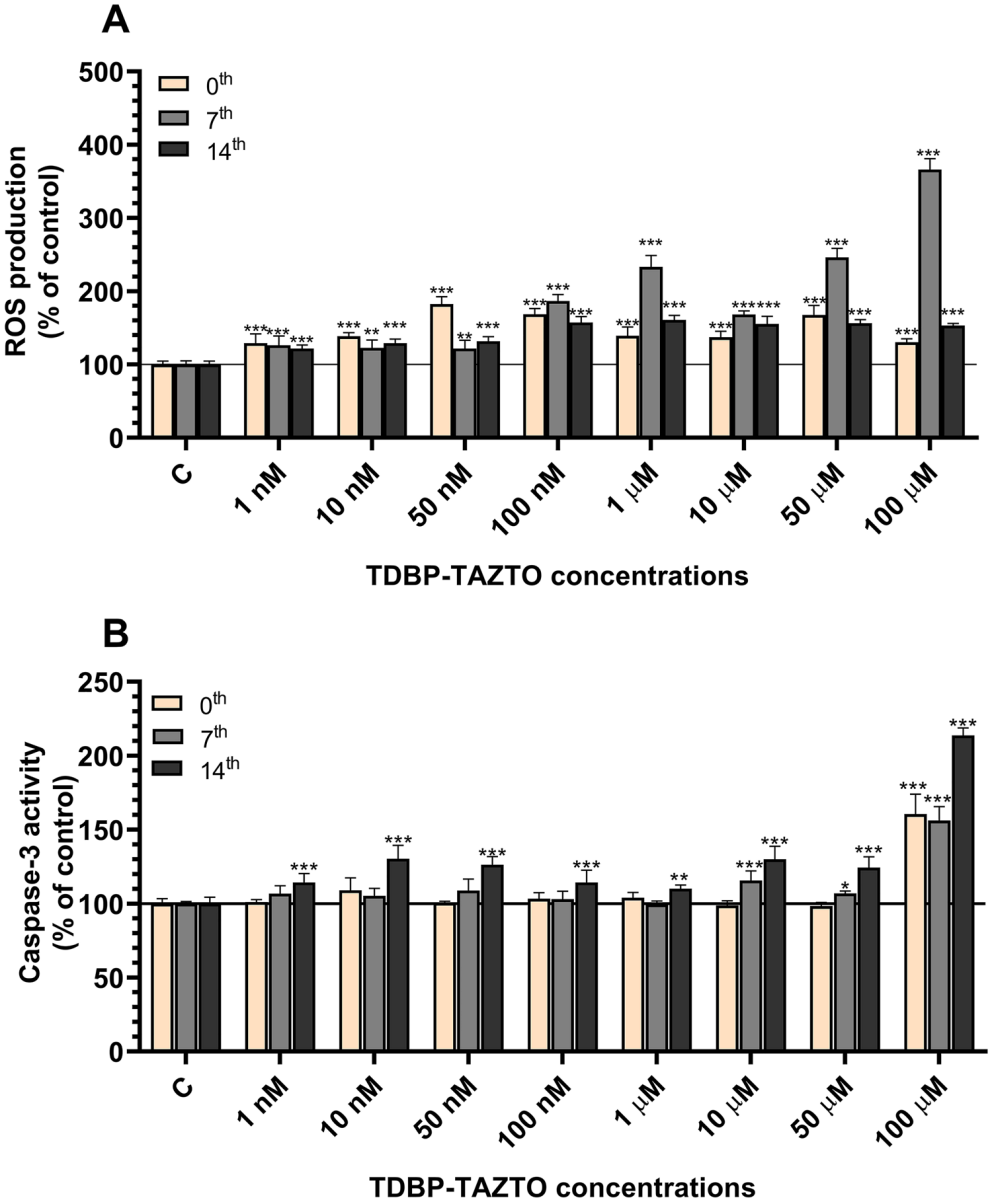
Effect of the increasing concentrations (1 nM–100 μM) of TDBP-TAZTO on ROS production **A** and caspase-3 activity **B** after 24-h exposure of undifferentiated (time 0) human neuroblastoma SH-SY5Y cells and those differentiated for 7 or 14 days. Data are expressed as mean ± SD of three independent experiments, each of which comprised six replicates per treatment group. *p < 0.05, **p < 0.01, ***p < 0.001 vs. control cells

Our results showed ROS production in the undifferentiated cells and those differentiated for 7 or 14 days after the 24-h exposure to TDBP-TAZTO at all studied concentrations (1, 10, 50, and 100 nM and 1, 10, 50, and 100 µM). In the undifferentiated cells, an increase in the range from 28.97 to 82.47% was observed, compared to the control (Fig. 2A). In cells differentiated for 7 days and exposed to TDBP-TAZTO for 24 h, the increase in ROS production was in the range from 21.72 to 266.04%, compared to the control. In cells differentiated for 14 days after the 24-h exposure to TDBP-TAZTO, there was a 21.58–60.69% increase in the ROS production, compared to the control (Fig. 2A).

#### Caspase-3 Activity

Our results showed that the 24-h exposure to TDBP-TAZTO in the undifferentiated SH-SY5Y cells increased caspase-3 activity only at the 100 µM concentration (a 60.57% increase, compared to the control) (Fig. 2B). In cells differentiated for 7 days and exposed to TDBP-TAZTO for 24 h, caspase-3 activity was increased at the concentrations of 10, 50, and 100 µM (an increase by 15.70, 6.88, and 56.19%, respectively, compared to the control) (Fig. 2B). In cells differentiated for 14 days after the 24-h exposure to TDBP-TAZTO, an increase in caspase-3 activity was observed at all tested concentrations (an increase in the range from 10.10 to 113.66%, compared to the control) (Fig. 2B).

### Cell Co-Treatment with the Agonist and Antagonist of AhR

Based on the resazurin reduction test, the concentration of 1 µM TDBP-TAZTO was determined as a toxic but not lethal concentration. Moreover, parameters that changed at the concentration of 1 µM TDBP-TAZTO at all tested time intervals (resazurin reduction and caspase-3 activity) were selected for the next analysis. Moreover, this experiment was aimed to determine the toxicity of the tool compounds (CAY10464 and βNF) alone and in co-treatment with 1 µM TDBP-TAZTO used in the subsequent analyses of enzymatic activity (EROD) and the molecular study (qPCR).

#### Resazurin Reduction Cell Viability Assay

**Fig. 3:**
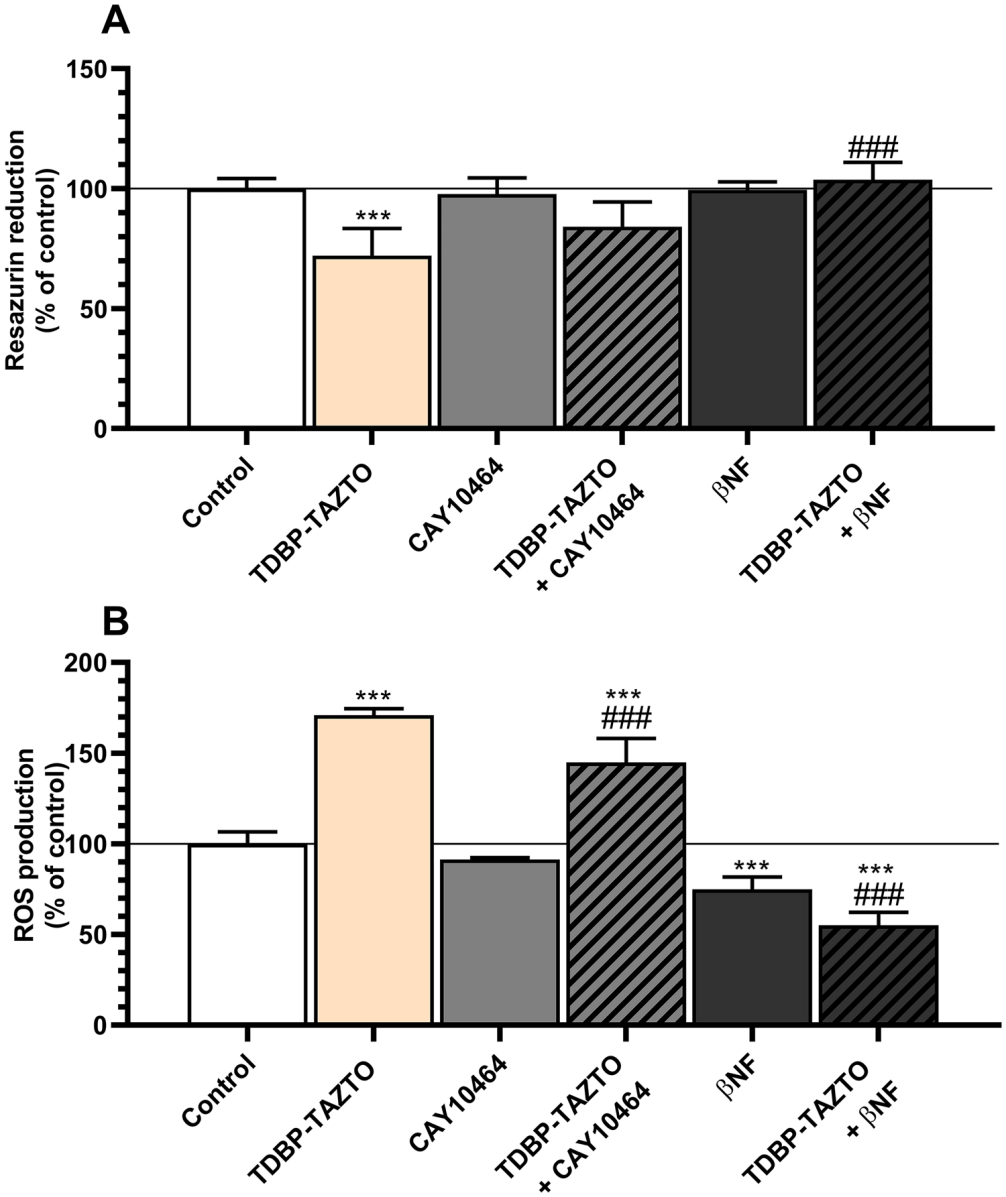
Effect of 1 μM TDBP-TAZTO, 1 μM CAY10464, and 1 μM βNF or co-treatment of TDBP-TAZTO with CAY10464 or βNF on for resazurin reduction assay **A** and ROS production **B**. Human neuroblastoma cell line SH-SY5Y cells differentiated for 14 days were exposed to the studied compounds for 24 h. Data are expressed as mean ± SD of three independent experiments, each of which comprised six replicates per treatment group. ***p < 0.001 vs. control cells; ###*p* < 0.001 vs. cells treated by TDBP-TAZTO alone

After 14 days of differentiation, the 24-h exposure to 1 µM TDBP-TAZTO decreased cell viability by 27.93%, compared to the control (Fig. 3A). Tool compounds CAY10464 and βNF at the concentration of 1 µM did not change cell metabolism significantly, compared to the control (Fig. 3A). The co-treatment of the cells with TDBP-TAZTO and CAY10464 or βNF did not change cell viability, compared to the control (Fig. 3A).

#### Reactive Oxygen Production (ROS)

After 14 days of differentiation, the 24-h exposure to 1 µM TDBP-TAZTO increased the ROS production by 71.03%, compared to the control. Tool compound CAY10464 did not change the ROS production, compared to the control (Fig. 3B). βNF decreased the ROS production by 25.13%, compared to the control (Fig. 3B). The co-treatment of the cells with TDBP-TAZTO and CAY10464 caused a 26.11% decline in the ROS production, compared to the TDBP-TAZTO-treated cells. A 44.95% decrease in the ROS production was recorded in cells co-treated with TDBP-TAZTO and βNF, compared to the vehicle control cells (Fig. 3B).

### Cytochrome P450 Activity: EROD Assay

**Fig. 4:**
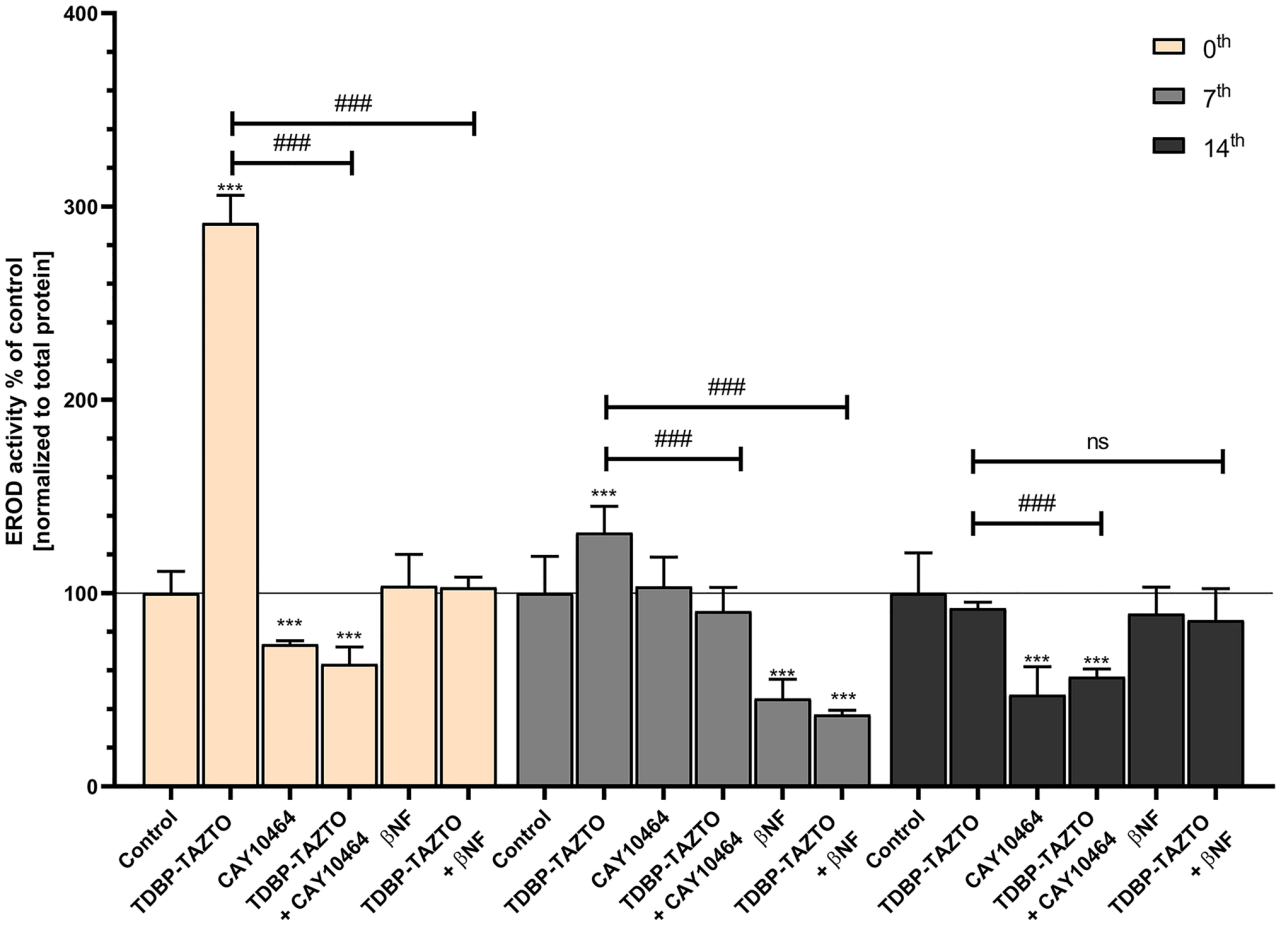
Effect of 1 μM TDBP-TAZTO, 1 μM CAY10464, and 1 μM βNF or co-treatment of TDBP-TAZTO with CAY10464 or βNF on the ethoxyresorufin-O-deethylase (EROD) assay. Undifferentiated (time 0) human neuroblastoma cell line SH-SY5Y cells and those differentiated for 7 or 14 days were exposed to the studied compounds for 24 h. Data are expressed as mean ± SD of three independent experiments, each of which comprised six replicates per treatment group. ***p < 0.001 vs. control cells; ###*p* < 0.001 vs. cells treated by TDBP-TAZTO alone

In the undifferentiated SH-SY5Y cells exposed to 1 µM TDBP-TAZTO, a 191.42% increase in the EROD activity was observed, compared to the control (Fig. 4). The 1 µM concentration of CAY10464 decreased the EROD activity by 26.43%, compared to the control cells (Fig. 4). Moreover, cells co-treated with CAY10464 and TDBP-TAZTO were characterized by decreased EROD activity by 36.66 and 228.08%, compared to the control cells and those exposed to TDBP-TAZTO alone, respectively (Fig. 4). The 1 µM solution of βNF did not change the EROD activity, compared to the control; however, cells co-treated with βNF and TDBP-TAZTO exhibited decreased EROD activity, compared to cells treated with TDBP-TAZTO alone (a decrease by 188.43%) (Fig. 4).

In the SH-SY5Y cells differentiated for 7 days upon the exposure to 1 µM TDBP-TAZTO, increased EROD activity was observed (an increase by 31.26%, compared to the control) (Fig. 4). The 1 µM concentration of CAY10464 did not change EROD activity, compared to the control cells (Fig. 4). However, the cell co-treatment with CAY10464 and TDBP-TAZTO decreased EROD activity by 40.55%, compared to the TDBP-TAZTO-treated cells (Fig. 4). βNF alone decreased the EROD activity by 54.53%, compared to the control (Fig. 4). The cell co-treatment with βNF and TDBP-TAZTO decreased the EROD activity, compared to the control cells and those treated with TDBP-TAZTO alone (a decrease by 60.81 and 94.07%, respectively) (Fig. 4).

On the 14th day of differentiation of the SH-SY5Y cells, the 1 µM concentration of TDBP-TAZTO did not cause any changes in the EROD activity (Fig. 4). 1 µM of CAY10464 decreased the EROD activity by 52.62%, compared to the control cells (Fig. 4). Moreover, the cell co-treatment with CAY10464 and TDBP-TAZTO also decreased the EROD activity by 43.27%, compared to the control cells (Fig. 4). No changes in the EROD activity were observed in cells treated with βNF alone and co-treated with βNF and TDBP-TAZTO (Fig. 4).

### Real-Time PCR Analysis of mRNAs Specific to Genes Encoding TUBB3, AhR, CYP1A1, CYP1A2, and CYP2B6

**Fig. 5:**
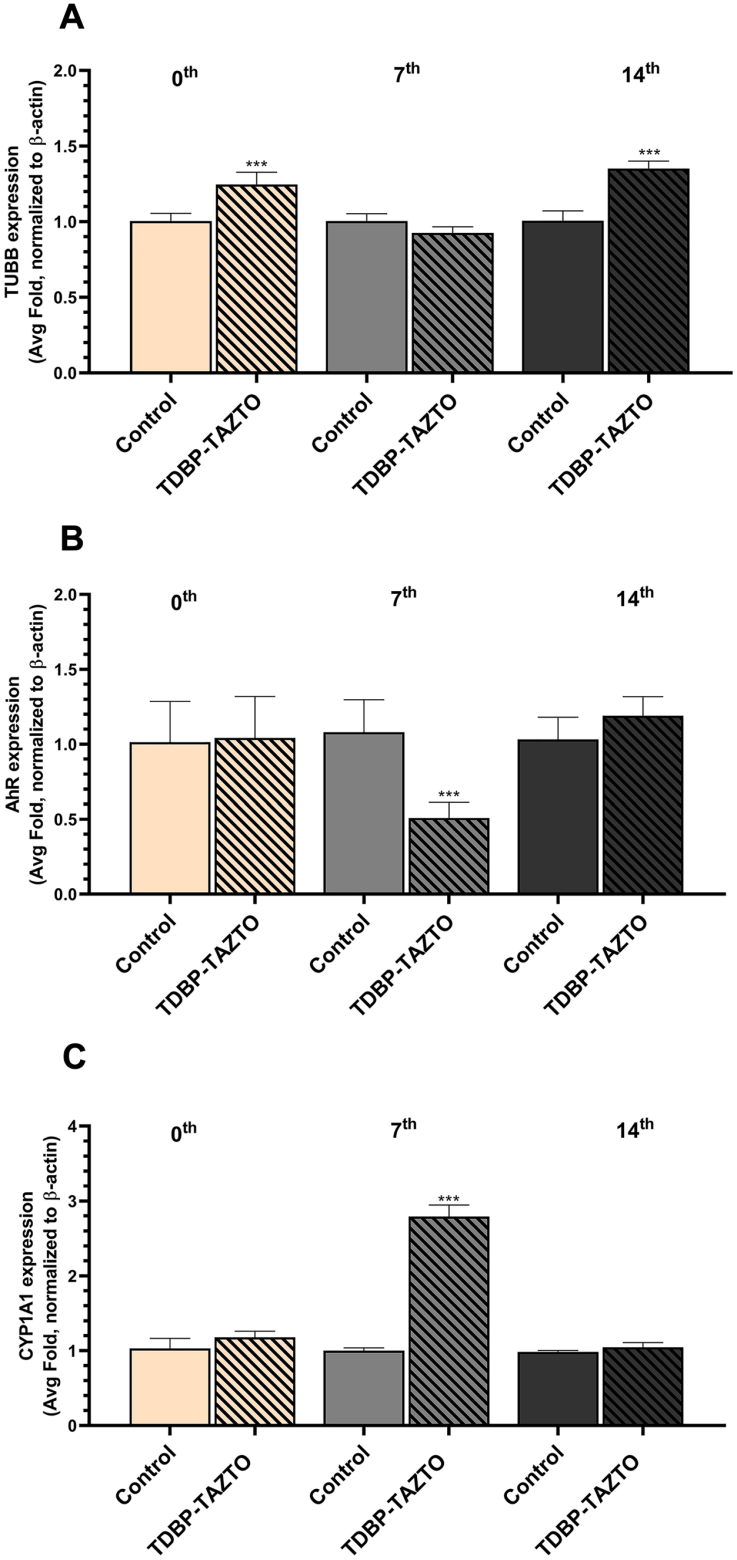
Effect of 1 µM TDBP-TAZTO on *TUBB3* **A**, *AhR* **B**, *CYP1A1* **C**, *CYP1A2*, and *CYP2B6* mRNA expression in undifferentiated SH-SY5Y cells (0 time) and those differentiated for 7 days (7th) and 14 days (14th) after 24-h exposure. mRNA expression was normalized to *ACTB*. No expression of *CYP1A2* and *CYP2B6* mRNA was detected in the experiments. Data are expressed as mean ± SD of three independent experiments, each of which comprised six replicates per treatment group. ***p < 0.001 vs. the vehicle control

In all the types of SH-SY5Y cells (undifferentiated, differentiated for 7 or 14 days), no mRNA expression of *CYP1A2* and *CYP2B6* was detected. Our experiments show that the 1 µM concentration of TDBP-TAZTO increased the *TUBB3* gene mRNA expression after 24-h stimulation of the undifferentiated SH-SY5Y cells (an increase by 24.67%, compared to the control) (Fig. 5A). In SH-SY5Y cells differentiated for 7 days, the 24-h stimulation with TDBP-TAZTO did not affect the *TUBB3* gene expression (Fig. 5A). Interestingly, a 35.17% increase in *TUBB3* gene expression was observed in cells differentiated for 14 days and exposed to TDBP-TAZTO for 24 h, compared to the control (Fig. 5A).

After the 24-h exposure to TDBP-TAZTO, changes in *AhR* gene expression were observed only in cells differentiated for 7 days (a decrease in the expression of *AhR* by 50.00%, compared to the control) (Fig. 5B). In SH-SY5Y cells that were undifferentiated or differentiated by 14 days, the 24-h exposure to TDBP-TAZTO did not change the expression of *AhR* (Fig. 5B).

Similarly, after the 24-h exposure to TDBP-TAZTO, there were changes in CYP1A1 gene expression only in the cells differentiated for 7 days (a 179.15% increase in the expression of *CYP1A1*, compared to the control) (Fig. 5C). In the undifferentiated SH-SY5Y cells or those differentiated for 14 days, the 24-h exposure to TDBP-TAZTO did not change the expression of *CYP1A1* (Fig. 5C).

**Fig. 6:**
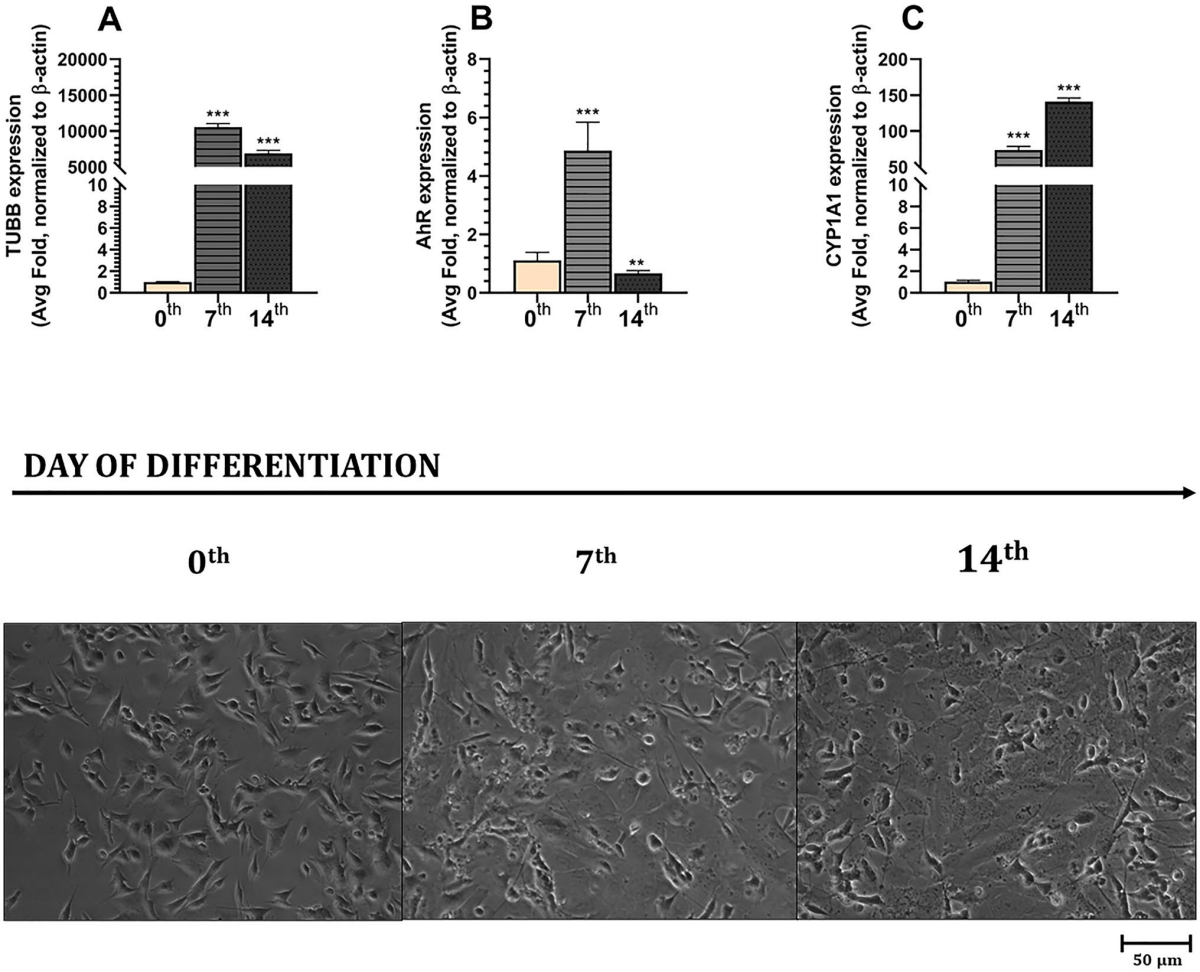
Changes in the time of the expression of *TUBB3* **A**, *AhR* **B**, *CYP1A1* **C**, *CYP1A2*, and *CYP2B6* mRNA in undifferentiated SH-SY5Y cells (0 time) and those differentiated for 7 days (7th) and 14 days (14th). mRNA expression was normalized to *ACTB*. No expression of *CYP1A2* and *CYP2B6* mRNA was detected in the experiments. The cells were visualized by using a microscope LSM 700, ZEISS. Data are expressed as mean ± SD of three independent experiments, each of which comprised six replicates per treatment group. **p < 0.01; ***p < 0.001 vs. the vehicle control

The experiments showed that the expression of the *TUBB3* gene increased by 1,054,259.00% in cells differentiated for 7 days and by 689,153.80% in cells differentiated for 14 days, compared to the undifferentiated cells (Fig. 6A). Moreover, during the differentiation process, the expression of the *AhR* gene increased by 386.58% in cells differentiated for 7 days and decreased by 33.35% in cells differentiated for 14 days, compared to the undifferentiated cells (Fig. 6B). During the differentiation process, the expression of *CYP1A*1 increased by 7356.73% in cells differentiated for 7 days and by 14,072.02% in cells differentiated for 14 days, compared to the undifferentiated cells (Fig. 6C).

## Discussion

As demonstrated by literature data, the concentration of xenobiotics in the environment is constantly increasing (Zhou et al. 2020). The reports show that the production of household materials such as electronics or plastic causes the release of many toxic substances, such as BFRs (Li et al. 2015). BFRs are able to cross the blood–brain barrier and accumulate in animals’ brains affecting their cells (Dong et al. 2015). Therefore, due to the emerging problem of environmental pollution, in this study, we have determined the impact of TDBP-TAZTO on the neuron differentiation process in vitro and its engagement in the toxic mechanism in such cells. Our results showed that, after the 24-h TDBP-TAZTO exposure of undifferentiated SH-SY5Y cells and those differentiated for 7 days, TDBP-TAZTO at all studied concentrations did not cause an increase in LDH release. In contrast, after the 24-h exposure of SH-SY5Y cells differentiated for 14 days, TDBP-TAZTO increase LDH release at all studied concentrations (1–100 nM and 1–100 µM), compared to the control. Interestingly, our results showed that TDBP-TAZTO at the concentrations of 1, 10, 50, and 100 µM decreased resazurin reduction in the undifferentiated cells and in cells differentiated for 7 and 14 days. There are only a few reports in the literature describing the in vivo and in vitro toxicity of TDBP-TAZTO (Ruan et al. 2009; Li et al. 2011; Dong et al. 2015), but our study is the first to use the LDH release parameter to assess the toxicity of the compound. To date, an in vivo experiment has demonstrated that TDBP-TAZTO at a concentration of 1 µg/mL was toxic to developing zebrafish (*Danio rerio*) embryos after 72 h of exposure (Li et al. 2011). In contrast, in vitro toxicological evidence suggests that TDBP-TAZTO is not toxic to human hepatocarcinoma (HepG2) cells (Ruan et al. 2009). On the other hand, in primary cultures, TDBP-TAZTO was shown to be toxic at concentrations of 5–10 µM in cultures of rat neurons developing from cerebellar granule cells, but not in mature cerebellar granule neurons (Qu et al. 2011). Such observation contradicts our studies on the SH-SY5Y cell line, where undifferentiated cells were less sensitive to TDBP-TAZTO than the differentiated ones. Dong et al. (2015) described that TDBP-TAZTO at concentrations of 12.5, 25, 50, and 100 µM decreased cell metabolism in SH-SY5Y neuroblastoma cells measured with the MTT assay (Dong et al. 2015). It should be noted that both the resazurin reduction assay and the MTT test are based on the rate of cell metabolism and have similar sensitivity (Rampersad 2012). Therefore, in this aspect, our data are consistent with the data described by Dong et al. (2015). Such observation is consistent with previous studies where SH-SY5Y cells became more sensitive to 6-hydroxydopamine toxicity during differentiation (Lopes et al. 2010). Summarizing, the present results indicate higher sensitivity of SH-SY5Y cells to TDBP-TAZTO during differentiation.

Our results showed that the 24-h exposure to TDBP-TAZTO in the undifferentiated cells and those differentiated for 7 and 14 days increased ROS production at all studied concentrations. The highest increase in ROS production was observed in cells differentiated for 7 days. Moreover, our results show that the 24-h exposure to TDBP-TAZTO in the undifferentiated SH-SY5Y cells resulted in an increase in caspase-3 production only at the 100 µM concentration. However, in cells differentiated for 7 days and exposed to the TDBP-TAZTO for 24 h, an increase in caspase-3 activity was observed at concentrations of 10, 50, and 100 µM. In cells differentiated for 14 days and exposed to TDBP-TAZTO for 24 h, the studied compound caused an increase in caspase-3 activity at all tested concentrations. Similarly, the apoptosis process induced by the presence of TDBP-TAZTO has been demonstrated in the hippocampus of adult rats given 5 or 50 mg/kg TDBP-TAZTO (Ye et al. 2015). Ye et al. (2015) reported an increase in the expression of caspase-3 and the Bax protein with a simultaneous decrease in the expression of the Bcl protein. Moreover, the authors postulated that TDBP-TAZTO induced apoptosis in rat hippocampus cells as a result of an increase in ROS production and inflammatory markers (Ye et al. 2015). A similar observation was described by Dong et al. (2015), where 12.5, 25, and 50 µM TDBP-TAZTO increased the expression of the Bax protein and decreased the expression of the Bcl-2 protein in the SH-SY5Y neuroblastoma cell line after 48 h of exposure (Dong et al. 2015). Moreover, these authors showed that TDBP-TAZTO induced DNA fragmentation at the same time interval. Dong et al. (2015) also observed an increase in the production of malondialdehyde (MDA) and superoxide dismutase (SOD) and a decrease in the amount of glutathione (GSH). Hence, the authors claim that the increase in ROS production initiates the apoptosis process.

AhR is a receptor involved in the mechanism of toxicity of many xenobiotics; however, the knowledge of TDBP-TAZTO and AhR during the neuron differentiation process in vitro is insufficient. Therefore, to determine the involvement of AhR in the toxic effect of TDBP-TAZTO in the SH-SY5Y cells, we conducted experiments with an AhR antagonist (CAY10464) and agonist (βNF). Our results showed that both tool compounds reduced the TDBP-TAZTO-induced ROS production. Moreover, CAY10464 and βNF increased the TDBP-TAZTO-reduced cell viability. This showed the abolition of the TDBP-TAZTO effect in the cells. Therefore, we suppose that the toxic effect of the tested compound is related to the AhR activity; thus, AhR is involved in the TDBP-TAZTO mechanism of action.

In our experiments, we showed that TDBP-TAZTO strongly increased EROD activity in the undifferentiated SH-SY5Y cells. Moreover, the AhR antagonist (CAY10464) prevented an increase in EROD activity caused by TDBP-TAZTO. Interestingly, the AhR antagonist (βNF) also prevented an increase in EROD activity stimulated by TDBP-TAZTO. In the undifferentiated cells, TDBP-TAZTO increased *TUBB3* gene expression but did not affect the expression of *AhR* and *CYP1A1* mRNA. During the differentiation process (day 7), the basal mRNA expression of *AhR*, *CYP1A1*, and *TUBB3* increased 5, 73, and 10,543 times, respectively, compared to the undifferentiated cells, which showed changes in the sensitivity of the tested cells to TDBP-TAZTO. Therefore, the TDBP-TAZTO-stimulated EROD activity was not as strong as in the undifferentiated cells. Similar as in the undifferentiated cells, CAY10464 and βNF inhibited changes in EROD activity induced by TDBP-TAZTO. Paradoxically, in cells differentiated for 7 days, the AhR agonist (βNF) decreased EROD activity much more potently than the AhR antagonist. Interestingly, in cells differentiated for 7 days, the treatment with TDBP-TAZTO decreased the *AhR* mRNA expression and strongly increased the *CYP1A1* gene expression, but no changes in the *TUBB3* mRNA expression were observed. Since the highest expression of *AhR* mRNA was observed on differentiation day 7, the use of the strong AhR (βNF) agonist could result in negative feedback reduction in the amount of CYP1A1 and, in consequence, reduction of EROD activity. This would explain the lowest EROD activity in the group co-treated with βNF and TDBP-TAZTO.

Finally, the highest mRNA expression of *CYP1A1* was observed in the SH-SY5Y cells differentiated for 14 days, compared to the undifferentiated cells. Moreover, after the cell exposure to TDBP-TAZTO, no changes in EROD activity were observed. CAY10464 alone and in the co-treatment with TDBP-TAZTO decreased EROD activity, compared to cells treated with TDBP-TAZTO. Interestingly, βNF or βNF in the co-treatment with TDBP-TAZTO did not affect EROD activity. The low activity of EROD may have been an effect of the high basal expression of CYP1A1 in cells and/or differentiation of the SH-SY5Y cells into neuronal cells. Moreover, in cells differentiated for 14 days and treated with TDBP-TAZTO, we did not observe any changes in the mRNA expression of the *AhR* and *CYP1A1* genes or an increase in the expression of the *TUBB3* gene.

As shown in literature, during differentiation, SH-SY5Y cells lose their ability to proliferate and gain the features and morphology of neuronal cells (Kovalevich and Langford 2013; Shipley et al. 2016). During the differentiation process, tubulin expression increases; therefore, its gene activity is regarded as an indicator of the success of neuron differentiation (Latremoliere et al. 2018). Such a phenomenon was observed in our research, which confirms the correct differentiation process of the SH-SY5Y cells. Moreover, our experiments also showed that the application of TDBP-TAZTO contributed to an increase in tubulin (*TUBB3* gene) expression in the undifferentiated SH-SY5Y cells and those differentiated for 14 days. At the time when the increase in the expression of *TUBB3* mRNA was observed, there were no changes in the expression of *AhR* and *CYP1A1* mRNA. It has been described that beta tubulin affects the AhR function via an Arnt-mediated mechanism in *Spodoptera frugiperda* pupal ovarian (Sf9) cells (Zhang et al. 2010). Further studies showed that G protein-coupled receptor 30 (GPR30) agonist G-1 increased AhR signaling by inhibition of tubulin assembly and cell cycle arrest in human breast adenocarcinoma (MCF-7) cells (Tarnow et al. 2016). Therefore, our results suggest that TDBP-TAZTO can disrupt and/or potentially increase the differentiation process by crosstalk of tubulin and AhR. To date, it has been described that AhR mediates zebrafish neurogenesis and gliogenesis, especially the differentiation of oligodendrocyte or Schwann cells (Wu et al. 2019). Moreover, Wu et al. (2019) showed that AhR agonists in neuroblastoma cells may induce differentiation. Similar results were reported by other teams where overexpression of AhR caused neural differentiation of Neuro2a cells (Akahoshi et al. 2006), and high expression of AhR was observed in samples from human neuroblastoma cells (Wu et al. 2014). Similar to our studies, an increase in *Cyp1a* mRNA expression was observed during differentiation in zebrafish brain embryos (Wu et al. 2019). Therefore, our data on the basal change in the mRNA expression of the amount of *TUBB3*, *AhR*, and *CYP1A1* in the differentiated neuronal cells is consistent with the current state of knowledge about neuronal cell differentiation.

Our paper is the first report on the role of TDBP-TAZTO in the induction of *TUBB3*, *AhR*, and *CYP1A1* mRNA expression and EROD activity. Moreover, we have proved that TDBP-TAZTO, i.e., a well-described environmental pollutant, acts through an AhR-dependent pathway, which is correlated with tubulin expression as well. Nevertheless, our results showed for the first time that the sensitivity to TDBP-TAZTO changes during the differentiation process, which is correlated with some crosstalk between TUBB3 and AhR. Unfortunately, there are no comparative data from other publications on the impact of TDBP-TAZTO on the aforementioned parameters. Therefore, more experiments need to be conducted in detail to determine the toxicity mechanism of this compound on the neuron differentiation process.

## Conclusion

Our experiment shows that, during the differentiation process, the ability of TDBP-TAZTO to induce EROD activity in SH-SY5Y cells decreased successively, which could be an effect of cell differentiation into neurons. The strongest induction of EROD activity was observed especially in the undifferentiated cells, whereas TDBP-TAZTO did not induce EROD activity after 14 days of differentiation. Moreover, our experiments show that, during differentiation, the ability of the AhR antagonist (CAY10464) and agonist (βNF) to affect EROD activity changed as well. Changes observed in EROD activity after stimulation with CAY10464 and βNF correlated with different *AhR* and *CYP1A1* mRNA expression during differentiation. The experiments showed that the protocol applied ensured effective differentiation of the SH-SY5Y cell line, which was confirmed by the high increase in *TUBB3* gene expression. Our experiments also show that no mRNA expression of *CYP1A2* and *CYP2B6* was detected in all types of SH-SY5Y cells (undifferentiated, differentiated for 7 or 14 days). Since no *CYP2B6* mRNA expression was detected, the CAR receptor may not be involved in the TDBP-TAZTO mechanism of action.

## Abbreviations

AhR: Aryl hydrocarbon receptor
CAR: Constitutive androstane receptor
DMEM/F12: Dulbecco’s Modified Eagle Medium/Nutrient Mixture F-12
DMSO: Dimethyl sulfoxide
EROD: Ethoxyresorufin-O-deethylase
FBS: Fetal bovine serum
H_2_DCFDA: 2′,7′-Dichlorofluorescin diacetate
ROS: Reactive oxygen species
LDH: Lactate dehydrogenase
EROD: Ethoxyresorufin-O-deethylase
βNF: β‐Naphthoflavone
PBS: Phosphate-buffered saline
TDBP-TAZTO: Tris (2,3-dibromopropyl) isocyanurate
